# Supplementary material for: Chromothripsis during telomere crisis is independent of NHEJ, and consistent with a replicative origin
Source: Genome Res. 2019 May;29(5):737–49. doi: 10.1101/gr.240705.118 (PMC6499312; doi:10.1101/gr.240705.118)
Supplement: Supplemental Material [file supp_gr.240705.118_Supplemental_file_1.zip › contigs/annotated_contigs/DB49/contig.2.DB49_length_871_mean_cov_16.0792192882.docx]

**DB49_length_871_mean_cov_16.0792192882**

```
CTTCTGTTTTTTGAGTTTTTGATTATGACCATT|AACTGAAGAAAGACAAACTGTAATAACTGCTTGAGATTAG|CAAACTGTCAAGTG
 >chr21:29458107-29458140 + E=6e-05                                       >chrY:5901612
AATATAGACAGCAATCTGCAGTTTGAAAATGCCAATCACTACAAATCACAGGAAAAACATCTGTGCTTCAGTGGAAACACTACATTGTT
1-59016918 + E=0e+00
TAGATCACACTCCTTCAGACTTTTGAGATAGGATCTCCATCACCCAGACTGGAATGCAAGGGTATGATCCTGGCTCACAGCAACCTCCA

CTTCCCTGGCTCAAGGGATTCTCCCACCTCAGCCTCCTGAGTAGCTGGGACTACAGGCTCATGCCATCATGCTCAGCTAATTTTTGGAA

ACTTTTTTTGTAGAGAGAGGGTCTATGTTGCTCAGGCTGGTCTCGAAATCCTGGGGTCAAGTGATCTGTCTACCTCAACCTCCCAAAGT

GCTGGGATAACAGGCGTGAGCCACTGCACCCAGCCCCTTTAAGGTTTTCTAGTCTAGCCTTTCACCTGTTTTATGGTATGTGAAGTTGA

AAAGAACTGATAATGCAAATGATTCTTGTTTATAAACATGCAAATCAACCTGCCTTGGAAAGCCAATCCTTCTCCATGTGGCAAAGCCA

CTTATGCATCAATTTCCAGTTTATTTCAATATTCTGAGTAATAGATGTGTCTGTCCCTTTGGGTTCCCTTTTGAACTGGTTGTAACATC

TCACTGCTCATTTCATTAATAAAATATTTAACATTTATATTTATATGAGTGATGGTTTTTCCTGTTTTTGAAAAGTTACCCTTTAACAA

ATATAACATGCACAGGTGAGTTAATGCCATGCTTCTCAAAGTATGGCCTGTGGAAGGAGCAGCCTGG|TGCTC
```
